# Supplementary material for: The Effectiveness of Different Concepts of Bracing in Adolescent Idiopathic Scoliosis (AIS): A Systematic Review and Meta-Analysis
Source: J Clin Med. 2021 May 15;10(10):2145. doi: 10.3390/jcm10102145 (PMC8156678; doi:10.3390/jcm10102145)
Supplement: Supplementary file 1 [file jcm-10-02145-s001.zip › jcm-1233145-supplementary.pdf]

SUPPLEMENTARY MATERIALS

META-ANALYSIS PAPERS

FIRST AUTHOR	YEAR	RISK OF BIAS	N. OF PATIENTS	COBB	RISSER	KIND OF BRACE	BRACE	FOLLOW-UP	SUCCESS/FAILURE RATE	PERCENTAGE
XU	2019	14 24	90	40-45	0-3	RIGID FULL TIME	BOSTON	1.3 Y	≤5°	51,10%
GRIVAS	2003	7 16	28	20-39	MENARCHE	RIGID FULL TIME	MODIFIED BOSTON		≤5°	82%
SAPETA	2010	7 16	79	20-45	0-4	RIGID FULL TIME	CHENEAU	1-5YEARS	≤5°	48%
MAUYAMA	2015	9 16	33	25-40	0-2/>1 Y POST-MEN	RIGID FULL TIME	RIGO-CHENEAU	MEAN 2,8	≤5°	76%
AULISA	2020	12 16	163	20-60 (MEAN 28,98)	0-4	RIGID FULL TIME	PASB	AT LEAST 10 Y	≤5°	65,60%
WEISS	2017	9 16	25	≥40	0-2	RIGID FULL TIME	GENISINGEN	1,5 Y	≤5°	92%
LAUTEUR	2017	10 16	142	10 25	0-3	RIGID NIGHT TIME	NOT STATED	3,75 Y	≤5°	83%
PRICE	1990	7 16	139	25-49	0-2	RIGID NIGHT TIME	CHARLESTON	>1 Y	≤5°	83%
LEE	2012	9 16	95	25-40	0-2/>1 Y POST-MEN	RIGID NIGHT TIME	CHARLESTON	>2 Y AFTER BRACE	≤5°	84,20%
ORTH-NISSEN	2016	7 16	63	25-40	0-2/>1 Y POST-MEN	RIGID NIGHT TIME	PROVIDENCE	MIN 2 Y	≤5°	57%
D'AMATO	2001	8 16	102	20-42	0-2	RIGID NIGHT TIME	PROVIDENCE	MIN 2 Y AFTER BRACE	≤5°	74%
SIMONY	2019	10 16	80	20-45	>1 Y POST-MEN	RIGID NIGHT TIME	PROVIDENCE	1 Y AFTER STOP	≤5°	89%
COILLARD	2007	8 16	170	25-40	0-2	SOFT FULL TIME	SPINECOR	2 AFTER STOP	≤5°	59,40%
COILLARD	2014	16 24	26	15-30	0-2	SOFT FULL TIME	SPINECOR	5 Y	≤5°	73%
			21			CONTROL				52,70%
YRJONEN	2006	12 24	36	>25	0-3	RIGID NIGHT TIME	PROVIDENCE	1,8 Y	≤5°	72%
			36	>25		RIGID FULL TIME	BOSTON			78%
GUO	2014	13 24	18	20-30	0-2/>1 Y POST-MEN	RIGID FULL TIME	NOT STATED	2 AFTER STOP	≤5°	94,60%
			20			SOFT FULL TIME	SPINECOR			65%

BUBBLE GRAPH ANALYSIS

AUTHOR	MINORS	SUCCESS	PT
XU ET AL 2019	8	51,10%	90
GRIVAS ET AL. 2003	7	82%	28
MARUYAMA ET AL. 2015	9	76%	33
SAPETA ET AL. 2010	7	48%	79
WEISS ET AL. 2017	9	92%	25
AULISA ET AL. 2020	12	65,60%	163
LAUTEUR EY AL. 2017	10	83%	142
PRICE ET AL. 1990	7	83%	139
LEE ET AL. 2012	9	84,20%	95
D'AMATO ET AL. 2001	8	74%	102
NISSEN ET AL. 2016	7	57%	63
SIMONY ET AL. 2019	10	89%	80
COILLARD ET AL. 2007	8	58,40%	170

SKELETAL MATURITY ASSESSMENT

Papers included

PAPER	N° PT	RISSER	SUCCESS
XU ET AL.	64	0-1	42,00%
XU ET AL.*	38	0-2	48%
MARUYAMA ET AL	33	0-2	76%
WEISS ET AL.	25	0-2	92%
PRICE ET AL.	139	0-2	83%
LEE ET AL.	95	0-2	84,00%
ORTH-NISSEN ET AL.	63	0-2	57%
D'AMATO ET AL.	102	0-2	74%
COILLARD ET AL.	170	0-2	58%
COILLARD (2ND) ET AL.	26	0-2	73%
GUO ET AL.	18	0-2	95%
GUO (S) ET AL.	20	0-2	65%
XU ET AL.*	46	0-3	51%
LAUTEUR ET AL.	142	0-3	83%
YRJONEN (N) ET AL.	36	0-3	78%
YRJONEN ET AL. *	36	0-3	72,00%
SAPETA ET AL.	79	0-4	48,00%
AULISA ET AL.	163	0-4	66%
